# The Relative Bioavailability of Lutein and Zeaxanthin in the Presence of Omega-3 Supplements and Their Effect on Oxidative Stress Levels in Humans: A Pilot Study

**DOI:** 10.3390/nu18121914

**Published:** 2026-06-12

**Authors:** Kingsley Arua Kalu, Charles McMonnies, Sophia Lin, Jayashree Arcot

**Affiliations:** 1 Food and Health, School of Chemical Engineering, University of New South Wales, Sydney, NSW 2052, Australia; j.arcot@unsw.edu.au; 2 School of Optometry and Vision Science, University of New South Wales, Sydney, NSW 2052, Australia; c.mcmonnies@unsw.edu.au; 3 School of Health Sciences, University of New South Wales, Sydney, NSW 2052, Australia; sophia.lin@unsw.edu.au

**Keywords:** lutein, zeaxanthin, omega-3 fatty acids, bioavailability, oxidative stress, iAUC, AUC, malondialdehyde, dietary supplementation, nutrient kinetics

## Abstract

**Introduction:** Lutein+Zeaxanthin (L+Z) are the major constituents of macular pigments of the retina. There is a lack of information on the bioavailability of the two compounds in the presence and absence of omega-3 fatty acids in L+Z supplements which are commonly prescribed to treat macular degeneration. Despite growing interest in L+Z supplementation, there remains a limited understanding of their short-term bioavailability dynamics and the potential added value of omega-3 co-supplementation. This pilot study reports on the bioavailability of serum responses to L+Z supplements in the presence of omega-3 fatty acids and evaluates time-resolved analytical approaches using Area Under the Curve. **Subjects/Methods**: A total of 10 men and six women with an average age of 31.38 ± 1.27 years participated in this randomised, non-blinded, controlled study for a total of 19 days (7-day wash-out period plus 12-day intervention period). The control group (n = 9) consumed the L+Z supplement (12 mg/d) only, while the intervention group (n = 7) consumed the L+Z supplement along with 900 mg/d of an omega-3 supplement (540 mg EPA + DHA 360 mg). Each group adhered to a comprehensive low-carotenoid and omega-3 diet list (LCOD) for the 7-day wash-out period and the 12-day intervention period. The participants reported the foods they consumed daily in their diet logbooks, online logs, and the ASA 24 diet assessment log over the study period. The body composition of each subject in the two groups was assessed before and after the study using a SECA body composition analyser, and the relative serum L+Z response in both groups was determined using Area Under the Curve (AUC and incremental AUC) by trapezoidal approximation. **Results:** The mean ± SEM baseline serum lutein+zeaxanthin (L+Z) concentrations measured at the end of the wash-out period (Day 7) were 2.23 ± 0.65 µg/mL in the control group and 1.20 ± 0.53 µg/mL in the intervention group. Following wash-out, serum L+Z concentrations increased in both groups, reaching 2.81 ± 0.90 µg/mL (control) and 2.63 ± 1.21 µg/mL (intervention) at Day 13, and 2.98 ± 0.69 µg/mL (control) and 3.02 µg/mL (intervention) at Day 19. Total exposure assessed by AUC_7–13_ and AUC_13–19_ did not differ significantly between the groups (*p* > 0.05). Incremental exposure analyses identified the post-wash-out period as the primary biologically responsive window, with higher mean incremental L+Z bioavailability in the intervention group (4.36 µg/day/mL) compared with the control group (3.00 µg/day/mL), although this difference was not statistically significant (*p* > 0.05). No significant effect of omega-3 co-supplementation on oxidative stress biomarkers was observed (*p* > 0.05). **Conclusion:** Omega-3 co-supplementation did not demonstrate a consistent additional benefit on L+Z bioavailability or oxidative stress markers. Day-resolved analyses using iAUC revealed temporal patterns not captured by conventional AUC measures. These exploratory findings should be interpreted with caution and confirmed in larger, longer-term studies.

## 1. Introduction

Many studies have been conducted on omega-3 fatty acids (supplementary and dietary), which mainly focus on the efficacies of eicosapentaenoic acid (EPA) and docosahexaenoic acid (DHA) because of their protective roles in many degenerative diseases [1,2]. In the last decade, landmark studies on lutein and zeaxanthin (L+Z) have been conducted on their role in age-related macular degeneration (AMD), which is the leading cause of blindness in developed countries [3,4,5,6]. Some of these studies investigated the effect of these carotenoids and omega-3 supplements on AMD prevention and progression [5,7,8]. However, controversies still exist regarding the role of omega-3 fatty acids on AMD prevention and progression [9,10]. While most studies have been conducted in patients with existing AMD conditions, fewer controlled trials [11,12] have been reported among healthy populations in relation to the bioavailability of L+Z. Nutritional supplements, particularly L, Z, and omega-3 fatty acids, have been of particular interest due to the decreasing quality of diets in many countries. Furthermore, there is an increasing interest in the complementary roles that supplements can play in enhancing bioavailability [13].

Also, L, Z, and omega-3s have been shown to play antioxidant roles that prevent oxidative retinal damage, which is a major risk factor in AMD pathogenesis [13]. In addition, increased visual acuity has been shown to be associated with these supplements, particularly among patients with the dry form of AMD [8]. Further investigation revealed that this observed effect is due to the synergy between omega-3 polyunsaturated fatty acids (PUFAs) and L, which leads to the accumulation of L in the blood and macula [13]. The rationale for increasing omega-3-PUFA levels is due to the observed high amount (50–60%) required by the outer segment photoreceptors, which implies that frequent turnover of these photoreceptors will necessitate continuous DHA supply [11]. An inadequate supply of fatty acids could contribute to the onset of AMD. The Rotterdam Study in 2167 individuals over a median 8.6-year period showed that an increase in dietary omega-3 intake reduces the genetic risk associated with AMD [14]. In addition to the protective roles of these compounds (L, Z, and omega-3-s), their dose response (together vs. alone) in relation to supplement intakes remains unclear [15]. However, responses to supplement dosing are variable and specific to individuals [15]. Hence, studies can only estimate a percentage relative response after intakes of a known amount of supplements.

The aim of this pilot study was to estimate the relative bioavailability of L and Z in the presence of omega-3 fatty acid supplements and to measure the oxidative stress levels in 16 healthy subjects provided with a commercially available supplement and to explore temporal patterns of biological responsiveness using both total and incremental approaches (AUC and iAUC). The hypothesis was that omega-3 fatty acid supplements combined with L+Z would be more bioavailable compared to taking L+Z supplements alone.

## 2. Materials (Subjects) and Methods

The study protocol and informed consent form were approved by the University of New South Wales Human Research Ethics Committee (HC No 210655) and registered as a clinical trial with the Australian New Zealand Clinical Trial Registry (ACTRN12622000837729, 15 June 2022).

Twenty-eight healthy subjects were recruited for the 19-day study that measured the relative bioavailability of L and Z in the presence of omega-3 fatty acid supplements as well as oxidative stress levels. The participants in the control group received commercially available supplements containing L+Z (10 mg + 2 mg), while the intervention group received the same L+Z formulation combined with concentrated omega-3 fatty acid providing eicosapentaenoic acid (EPA) (540 mg) and docosahexaenoic acid (DHA) (360 mg); all the supplements were sourced from the same batch. The effects of supplementation in both groups were analysed using mixed-effects models and the trapezoidal rule to estimate AUC and iAUC [16,17].

### 2.1. Subject Recruitment and Screening

These subjects completed the preliminary screening process that included anthropometry measurements using Bioimpedance (BIA platform seca, mBCA 555), completed a questionnaire on their background information (e.g., age, gender status, ethnicity, family history of AMD, bowel habits, atrophic, cardiovascular, pancreatic disorders, diabetes, and gastrointestinal diseases, current or recent supplement use, smoking status, alcohol intake, current or recent use of medications that may affect lipid absorption, and whether they were on any medically related special diet). Eight subjects withdrew before the end of the study and were not included in the final analysis. Hence, a total of sixteen subjects completed the study.

### 2.2. Dietary Logs, Assessments, and Wash-Out

The subjects completed pre- (prior to a 7-day wash-out) and post-dietary assessments during the 12-day intervention period using daily food records (via an online form and logbooks) and 24 h dietary recalls. Venous blood samples (20 mL) were collected at four time points: baseline, after the wash-out period (pre-intervention), mid-intervention, and at the end of the intervention. All the subjects were provided with a list of low-carotenoid foods (<6 mg L+Z) and low-omega-3 foods (<300 mg), constituting a low-carotenoid, omega-3-restricted diet (LCOD), and were instructed to adhere to this diet throughout both the wash-out and intervention phases. This approach was used to minimise dietary intake of L+Z and omega-3 fatty acids, thereby allowing a more accurate assessment of the effects of omega-3 supplementation on serum L+Z concentrations.

The LCOD list was developed from the USDA Food Composition Database (https://fdc.nal.usda.gov/) and Macular Foundation Australia’s menu list (https://www.mdfoundation.com.au/download-your-free-macula-menu/, 25 March 2020). This list had two parts: foods to consume and avoid during the study period. The list of foods to consume had about 106 low-carotenoid and low-omega-3 foods, while the list to avoid had 39 different carotenoid- and omega-3-rich foods. Adherence to the diets was monitored through weekly review of subjects’ logbooks (hard and online copy), including a weekly communication via a telephone call (once) for any adverse effect associated with excessive intakes (rare) during the intervention phase.

### 2.3. Randomization and Post-Assessment

The study subjects were randomly (non-blinded) allocated to either the control (L+Z supplements only) or the intervention group (L+Z, plus omega-3 supplements [EPA+DHA]). Randomisation was performed using GraphPad software Prism 8.4.0 [18]. Following the wash-out phase, the subjects were monitored over a 12-day period for any associated changes. All the subjects were provided with commercially obtained supplements in separate amber-coloured bottles at the beginning of the intervention, to be consumed daily over a 12-day period during the study. The subjects were provided with instructions on the number of capsules to take daily (the control group consumed 1 capsule of the L+Z supplement daily, while the intervention group consumed L+Z (1 capsule/day) plus omega-3 supplements (1 capsule/day). Adherence to both the LCOD and supplement protocols were monitored. The foods consumed during the trial from the LCOD list were recorded (online and hardcopy) by the subjects in their logbook (daily), and each subject completed a 24 h dietary recall on Days 0, 7 (wash-out), and 13 and 19 (intervention) during the study. Compliance was assessed by counting the remaining capsules in each subject’s supplement bottle at the end of the intervention phase. Venous blood samples were collected and processed on the same day (Days 0, 7, 13, and 19) with serum and red blood cells (RBCs) stored separately at −80 °C until further analysis.

### 2.4. Dietary Adherence Scores

Dietary adherence to the LCOD by the subjects was determined by assigning total adherence scores of 3/day for 19 days. These scores were developed for this study, but a similar approach has been used in the other literature [19]. For the wash-out stage, the level of adherence was determined by the difference in serum L+Z (before and after wash-out).

### 2.5. Specimen Collection and Analysis

A total of 20 mL of fasting venous blood samples (each for serum and RBC) were collected before and after wash-out (Days 0 and 7) and supplementation (Days 13 and 19). For serum collection, the samples were collected into a serum separator tube, inverted 8–10 times and allowed to sit upright for at least 15–30 min at room temperature to allow the blood to clot. The collected blood samples were then centrifuged at 1000× *g* for 15 min at room temperature to separate the serum which was recovered using a transfer pipette. Similarly, RBCs from the venous blood were centrifuged at 1500× *g* for 20 min at 4 °C, then plasma and the buffy coat were removed, and the RBC pellets were washed 3× with phosphate-buffered saline (PBS) and stored at −80 °C until further analysis [20]. All the blood samples were collected and processed on the same day. L and Z were determined in the serum, while omega-3 fatty acids were determined in the RBC pellets.

### 2.6. Lutein+Zeaxanthin and Omega-3 Fatty Acid Quantification

Serum L and Z levels in the subjects were determined by HPLC according to a described method [21,22,23,24] with slight modification. The injection volume was increased to 20 µL to enhance the separation of the two compounds and elution using an isocratic mode. The pooled serum was spiked with different concentrations (10, 20, 30 µL) of the working solution standards to test for extraction efficiency. Pure standards (L+Z) were injected 10× from a single vial to test for repeatability; the coefficient of variation (CV%) was 6.6%. The omega-3 fatty acids in the RBCs were determined using the Alqarni–McIntyre [25] method. For the RBCs, 50 µL was used instead of 20 µL due to difficulty in pipetting lower volumes. In addition, the RBCs were diluted 3× with PBS to ensure easy pipetting. Achieving a final pH of 4–5 for all the samples improved RBC extraction efficiency and recovery. All the extractions and analyses were carried out on the same day in replicates. The data obtained were processed using Mass Hunter quantitative and qualitative software (version 13.0).

### 2.7. Estimation of MDA Levels as an Oxidative Stress Biomarker

The (Malondialdehyde) MDA levels in the serum were determined using a lipid peroxidation assay kit (Product No: ab118970) supplied by BioVision (an Abcam company, Milpitas, CA, USA). Briefly, thiobarbitaric acid (TBA) reacts with the MDA present in serum in an acidic medium to form MDA-TBA product at 95 °C for 60 min. The absorbance of this product was determined colorimetrically at 532 nm (OD) using a spectrophotometer (SPECTROstar-601-0669, BMG LABTECH GmbH, Ortenberg, Germany), and the MDA levels were calculated using the kit-provided formula provided below [25].

B = (A_corrected_ − (y − intercept)/Slope)

MDA Concentration = B/V × 4* × D

B = Amount of MDA in sample calculated from the standard curve (in nmoles);

V = Original plasma volume used (in mL);

4* = Correction for using 200 µL of the 800 µL reaction mix;

D* = Sample dilution factor if the sample is diluted to fit within the standard curve range, or not all of the samples used in the MDA-TBA adduct step (prior to reaction well set up).

### 2.8. Data Management and Analysis

Subjects who missed even a single visit or did not adhere to the LCOD list of foods were not included in the study. The data were analysed using Excel, GraphPad and SPSS software version 26.0. The significance assessment was at a 2-tailed alpha level of 0.05. The AUC and iAUC for serum L+Z concentrations were calculated using the trapezoidal method [17]. AUC represented total serum L+Z over time, while iAUC quantified changes above baseline. Baseline concentrations were defined as Day 0 for the wash-out period (Days 0–7), Day 7 for the supplementation (intervention) period (Days 7–13), and Day 13 for the post-intervention period (Days 13–19), where visit 1 = Day 0, visit 2 = day 7, visit 3 = day 13 and visit 4 = day 19. Concentrations below baseline were set to zero before iAUC calculation. AUC and iAUC values are reported in µg·day/mL and calculated at the individual level prior to deriving group-level means. The data were reported as mean and standard error of the mean for each group. Accordingly, both metrics were reported to provide complementary interpretations. The formula for AUC = e.g., (C0 + C7)/2 × (D7 − D0) while iAUC = (ΔC0 − ΔC7)/2 × (D7 − D0). C represents serum L+Z concentration on the day of the visit, and D is the study day, while ΔC_t(day)_ = C_t(day)_ − C_baseline_. Sample preparation and analysis were done in duplicates.

## 3. Results

### 3.1. Participant Characteristics

Baseline characteristics are described in Table 1 (men and women) and Table 2 (control and intervention). All the subjects had no recent history of smoking, alcohol consumption or dietary supplement intake and were healthy. Most subjects were of African descent (50.0%), while 31.3% and 18.8% were from South-East Asia and other continents. The mean weight, height, body mass index (BMI), waist circumference, fat-free mass index (FFMI), total energy expenditure (TEE), and resting energy expenditure (REE) were significantly higher (*p* < 0.05) among men compared to women (Table 1), while women had significantly higher mean hip circumference (*p* < 0.05) (Table 1). The control group had significantly higher mean fat mass index (FMI) compared to the intervention group, while the mean differences observed across all other factors were not significant (*p* > 0.05) (Table 2). The % mean dietary adherence to the LCOD showed significant correlation with MDA levels at baseline (*p* < 0.05). Similarly, serum L+Z concentration at baseline showed significant correlation with the MDA concentration (*p* < 0.05) (Table 3). Women had a slightly higher mean % dietary adherence (88.0 ± 3.96%) than men (87.0 ± 3.33%), but the difference was not significant. All the subjects completed the 19-day visit, adhered to the LCOD list, and provided blood samples at each visit (four visits in total).

### 3.2. Serum Lutein and Zeaxanthin Concentrations Across Study Visits

Mean (±SEM) serum L+Z concentrations for the control and intervention groups across study visits (Days 0, 7, 13, and 19) are summarised in Table 3. Baseline serum L+Z concentrations were comparable between the groups, and absolute serum L+Z concentrations remained broadly similar between the groups. Also, Table 4 shows the absolute change from baseline for the control and intervention groups.

### 3.3. Area Under the Curve (AUC) and Incremental AUC

Total serum L+Z concentration was assessed using AUC/iAUC and was comparable between the control and intervention groups across study periods (Table 5, Table 6 and Table 7). AUC values for Days 0–7, Days 7–13, and Days 13–19 did not differ significantly between groups, indicating similar levels during this visit. Incremental AUC was determined using interval-specific baseline definitions: Day 0 for Days 0–7, Day 7 for Days 7–13, and Day 13 for Days 13–19. Individual-level (iAUC) values were calculated prior to the derivation of group-level means (Table 5 and Table 6).

### 3.4. Wash-Out (Days 0–7)

During the wash-out phase (Days 0–7), mean serum L+Z concentrations were monitored to characterise baseline serum L+Z status and inter-individual variability prior to the intervention phase (post-wash-out changes). The control group (n = 9) had mean serum L+Z concentrations that remained stable between Day 0 and Day 7, whereas the intervention group (n = 7) decreased over this period. Measurements on Days 13 and 19 post-wash-out are descriptive and serve only to illustrate subsequent temporal changes (Table 7).

**Table 7:** Serum lutein+zeaxanthin (L+Z) concentration of AUC and iAUC (control vs. intervention).

Outcome	Control (n = 9)	Intervention (n = 7)	*p*-Value †	Cohen’s d (95% CI)
AUC_0–7_ (µg·day/mL)	15.59	12.90	0.69	0.21 (−0.77, 1.18)
AUC_7–13_ (µg·day/mL)	15.12	11.49	0.58	−0.29 (−1.28, 0.71)
AUC_13–19_ (µg·day/mL)	17.39	16.94	0.96	0.03 (−0.93, 0.99)
iAUC_0–7_ (µg·day/mL)	1.62	0.01	0.10	0.81 (−0.22, 1.84)
iAUC_7–13_ (µg·day/mL)	3.00	4.36	0.68	0.23 (−0.76, 1.22) *
iAUC_13–19_ (µg·day/mL)	1.91	2.08	0.91	0.06 (−0.93, 1.05)

† *p*-values represent between-group comparisons (independent samples *t*-test). * Indicates a small-to-moderate effect size.

### 3.5. Early Phase (Days 7–13)

Using Day 7 as the reference baseline, the incremental iAUC analysis identified the early post-wash-out phase (Days 7–13) as exhibiting the greatest changes (Table 8). Mean iAUC values during this interval were higher in the intervention group compared with the control group, indicating a greater incremental change in serum L+Z concentrations relative to Day 7. Although the between-group difference did not reach statistical significance, the direction and magnitude of the effect suggest a biologically meaningful early response following wash-out.

### 3.6. Late Phase (Days 13–19)

When Day 13 was used as the baseline for the late phase (Days 13–19), iAUC values showed smaller between-group differences than in the early phase (Table 8). Incremental increases in serum L+Z concentrations were modest and similar in magnitude between the control and intervention groups, suggesting attenuation during this period. Similarly, the AUC values for Days 13–19 did not differ, indicating comparable total serum L+Z levels in this phase (Table 8). This suggests that the initial post-wash-out response observed between Days 7 and 13 had no further pronounced incremental changes during this period.

Overall, the wash-out phase effectively reduced baseline variability, particularly in the intervention group, as evidenced by a decline in circulating levels between Day 0 and Day 7. Following supplementation, both groups exhibited increases in serum L+Z concentrations, with comparable final levels by Day 19, suggesting no clear additional effect of omega-3 co-supplementation on overall bioavailability.

### 3.7. Absolute Changes in Oxidative Stress and Lipid Biomarkers

Absolute changes in oxidative stress (measured as serum MDA levels) and lipid biomarkers (RBC omega-3 fatty acids from baseline to the final study period) are summarised in Table 9. The biomarker values at baseline were obtained during the wash-out phase (Days 0–7), and absolute changes were calculated as differences from baseline.

### 3.8. Malondialdehyde (MDA)

In the control group, mean serum MDA concentrations showed a small absolute decrease from baseline to the final study period (−0.03µmol/L). In contrast, the intervention group exhibited an absolute increase in serum MDA levels over the same period (+0.09µmol/L) in Table 9. These changes indicate divergent oxidative stress trajectories between groups. However, given the magnitude of observed change and variability, the MDA outcomes were considered descriptive rather than primary inferential endpoints and suggest no clear advantage of omega-3 co-supplementation.

### 3.9. Omega-3 Polyunsaturated Fatty Acids (PUFAs)

Mean RBC omega-3PUFA levels increased modestly in both groups between baseline and the final study period (Table 9). The control group demonstrated a larger absolute increase (+1.21%), while the intervention group showed a smaller increase (+0.48%). These changes suggested modest alterations in omega-3 status over time.

Overall, absolute changes indicate small-to-moderate variations in oxidative stress and omega-3 status across the study period. These measures indicate biological variability, and absolute changes were interpreted descriptively and used to provide biochemical context. Primary biological interpretation focused on baseline-adjusted incremental changes (iAUC) and correlation analysis suggests no clear advantage of omega-3 co-supplementation.

### 3.10. Association Between Baseline Serum L+Z, Dietary Adherence and Oxidative Stress

Pearson’s correlation analysis was conducted to examine the associations among the serum L+Z concentrations, percentage dietary adherence, and MDA levels measured on Day 7 (end of wash-out). The results are summarised in Table 10.

A moderate positive correlation was observed between baseline serum L+Z concentrations and serum MDA levels at Day 7 (r = 0.529, *p* = 0.035). Similarly, percentage dietary adherence was moderately positively associated with MDA levels at Day 7 (r = 0.550, *p* = 0.027). Both associations were statistically significant using two-tailed tests.

These findings indicate that individuals with higher baseline serum L+Z status or greater dietary adherence tend to exhibit higher MDA concentrations at the end of the wash-out period. Given the exploratory nature of this analysis and the modest sample size (N = 16), the correlation results were interpreted as associative, with the intention of providing a mechanistic context.

## 4. Discussion

As a pilot bioavailability study, the primary aim was not to establish definitive efficacy, but rather to characterise absorption dynamics and explore methodological approaches for detecting biological responsiveness.

Therefore, the temporal/incremental changes in serum L+Z concentrations, oxidative stress, and omega-3 PUFA status, as well as their interrelationships, were examined across the wash-out period and the subsequent intervention/follow-up phases.

Rather than relying solely on single time-point comparisons, this study employed interval-specific analyses using iAUC to better capture biological responsiveness. While total serum L+Z concentrations, assessed by AUC, were comparable between groups across the study duration, the iAUC analysis revealed day-dependent differences that were not evident using AUC alone.

Also, no between-group differences in AUC/iAUC reached statistical significance (*p* > 0.05). Therefore, the “biological responsiveness” should be considered descriptive in nature and interpreted within the context of the pilot study design. This distinction (AUC and iAUC) is well established [26,27,28], in which iAUC is used to isolate changes relative to the baseline when baseline variability is substantial. In this present study, iAUC proved more informative than AUC for capturing dynamic physiological responses following wash-out.

The wash-out phase (Days 0–7) was used to establish the baseline for serum L+Z status before interpreting post-wash-out responses. Serum L+Z concentrations remained stable in the control group but declined in the intervention group over this period, highlighting meaningful inter-individual variability in serum L+Z status (Table 7). Controlled depletion studies have demonstrated that serum L+Z decline slowly, but short-term dietary restrictions can still produce measurable changes in circulating levels, depending on the baseline stores and metabolic turnover [29,30]. These findings underscore the relevance of defining baselines contextually and justify the use of Day 7 as a reference point for incremental analysis in this study.

The early post-wash-out phase (Days 7–13) was characterised by a greater increase in serum L+Z concentrations in the intervention group than in the control group (Table 8). This response is consistent with carotenoid absorption and transport kinetics involving micellar incorporation, lipoprotein-mediated transport and tissue redistribution [31,32].

Reviews of carotenoid bioavailability and human kinetics emphasise that measurable systemic changes occur days after dietary transitions, particularly for carotenoids such as L and Z [30,31,32]. Overall, the wash-out phase effectively reduced baseline levels, particularly in the intervention group, indicating successful minimisation of prior dietary influence. Following supplementation, both groups showed similar increases, with comparable final L+Z concentrations, suggesting no clear additional effect of omega-3 co-supplementation on overall bioavailability.

The identification of a discrete early response window underscores the importance of timing outcome measurements, and studies relying on later time points may fail to capture transient yet biologically relevant changes.

Day 13 was used as a baseline for late-phase analysis (Days 13–19), in which incremental differences between the groups diminished, suggesting stabilisation of circulating serum L+Z concentrations. Such plateauing effects are well documented in carotenoid supplementation and dietary intervention studies, where prolonged intakes result in saturation of transport and tissue pools rather than continued linear increases [32,33,34]. This pattern supports the interpretation that the early post-wash-out phase represents a period of greatest responsiveness.

Absolute changes in oxidative stress (measured via serum MDA) and lipid (RBC omega-3 fatty acids) biomarkers were examined descriptively (Table 9). Also, the reason for assessing the effect of omega-3 fatty acids in RBCs is based on scientific evidence linking RBC omega-3-PUFAs to AMD risk and overall retinal health [13,35,36,37]. The intervention group exhibited a greater absolute increase in serum MDA levels relative to the control, while omega-3-PUFA levels increased modestly in both groups. However, these findings were not treated as primary outcomes, as short-term changes in oxidative stress biomarkers can reflect transient metabolic flux and may not reliably indicate sustained/clinically relevant physiological effect [38]. Hence, further validation measurements are required over a long study period.

MDA is an end product of PUFA oxidation and has become a reliable oxidant stress marker in relation to lipid oxidation in many studies [39,40,41]. Serum MDA levels were used as a marker of lipid peroxidation activity rather than cumulative oxidative injury, and transient increases have been observed during dietary transitions/supplementation and postprandial metabolic adjustment [38,42,43].

Systematic reviews indicate dietary patterns do not uniformly reduce MDA at all time points, particularly for short-duration/small-sample interventions [42,43,44]. The assessment of lipid peroxidation is limited by the variability in methods, and no single method has been satisfactory. This study is limited to using a single assessment method, and future studies using more than one method will be necessary to further validate the observed trend. Accordingly, these absolute biomarker changes were interpreted cautiously and used to provide biochemical context rather than infer efficacy.

Correlational analysis indicates moderate positive associations between baseline serum L+Z concentrations and serum MDA levels at Day 7, as well as between dietary adherence and serum MDA (r ≈ 0.53–0.55) (Table 10). However, these analyses were exploratory and not adjusted for multiple comparisons. Given the small sample size, these findings should be interpreted as hypothesis-generating, and no causal inferences can be made. Confirmation in larger, adequately powered studies is required.

During the wash-out period, the subjects were instructed to adhere to the LCOD, which is low in carotenoid-rich foods (e.g., green leafy vegetables, coloured vegetables, eggs) and omega-3 fatty acids, both of which are known contributors to antioxidant capacity and membrane stability [45,46]. However, when the dietary supply of L+Z is acutely restricted, individuals with higher baseline tissue and circulating stores may undergo substantial metabolic mobilisation and turnover, which can coincide with increased lipid peroxidation markers, such as MDA [29,32,42].

This interpretation is consistent with evidence indicating that short-term dietary restriction can produce transiently elevated oxidative stress biomarkers, even in the presence of an antioxidant-rich baseline status [29,38,42].

The association between dietary adherence and serum MDA levels can be interpreted through the lens of the wash-out diet composition. Higher adherence during wash-out reflects greater compliance with the LCOD, which may have reduced antioxidant buffering capacity at a time when endogenous redox systems were adjusting [29,42,43]. Also, omega-3 fatty acids status has shown to exert indirect antioxidant and anti-inflammatory effects. However, low omega-3s availability has been associated with increased susceptibility to lipid peroxidation under conditions of metabolic stress [47,48]. Consequently, individuals most adherent to wash-out dietary restrictions may have reflected a transient change in oxidative status as revealed in the elevated serum MDA at Day 7, although this observation is exploratory and should be interpreted with caution (Table 10). These correlational findings provide a mechanistic baseline for carotenoid/serum L+Z status and behavioural dietary adherence, which modulates the magnitude of oxidative biomarker changes during the dietary restriction (wash-out) phase. These observations further underscore the value of the day-resolved, baseline-adjusted analysis (iAUC), which captures biological responsiveness at post-wash-out (Days 7–13 and Days 13–19).

Overall, these relationships should be interpreted as associative and exploratory, indicating the interplay between dietary composition, baseline carotenoid stores, and short-term oxidative responses. Future studies incorporating repeated oxidative stress measurements using a two-method approach (as validation) are necessary to determine sustained antioxidant outcomes.

Hence, the observed post-wash-out response is consistent with carotenoid absorption and redistribution dynamics rather than an omega-3-mediated antioxidant effect. This finding is consistent with previous studies demonstrating the synergistic role of omega-3 fatty acids in improving carotenoid absorption and transport, as well as their established protective effects on ocular health and a range of degenerative conditions, including age-related macular degeneration and neurodegenerative disease [45,46,47]. Elevated circulating concentrations of L+Z are known to support retinal health by increasing macular pigment density and improving resistance to oxidative and photic stress [48].

Nevertheless, the relatively short study duration and modest sample size limit the ability to draw definitive conclusions regarding the downstream effects of systemic oxidative stress and RBC omega-3 status. The observed post-wash-out response (AUC and iAUC) likely reflects the slower absorption and tissue redistribution of carotenoids, rather than a direct antioxidant effect mediated by omega-3 fatty acids.

Additionally, the relatively small sample size (n = 16) and absence of an a priori power calculation reflect the exploratory (pilot) nature of this study and necessitate cautious interpretation of the findings. Furthermore, the study was randomised but non-blinded, which may introduce potential bias.

Baseline imbalances between the groups were also observed. Specifically, serum L+Z concentrations at Day 7 were higher in the control group compared to the intervention group (2.23 vs. 1.20 µg/mL), and FMI differed significantly between the groups (control, 8.30 vs. intervention, 6.21 kg/m^2^, *p* = 0.038). These differences may confound the interpretation of the iAUC and between-group comparisons. Also, the relatively short duration of the study (12 days) limits interpretation to early circulating responses and does not permit conclusions regarding longer-term outcomes such as retinal deposition, macular pigment optical density changes, or sustained modulation of oxidative stress.

## 5. Conclusions

This pilot study investigated whether the intake of L+Z supplements, with or without omega-3 supplementation, provides additional benefit (bioavailability and oxidative stress levels). The addition of omega-3 supplementation did not demonstrate a consistent additional effect on MDA or other oxidative biomarkers during the study period. Despite the pilot nature of this study, including its relatively small sample size and short duration, the findings suggest that day-resolved, baseline-adjusted analyses, particularly using iAUC, may provide additional insight into patterns of biological responsiveness that are not captured by single time-point or total exposure (AUC) comparisons. However, these observations should be interpreted with caution and considered exploratory, warranting confirmation in larger and longer-term studies.

Although not statistically significant, the identification of Days 7–13 as the primary biologically responsive window with the greatest increase in the intervention group has important implications for study design, suggesting that sampling strategies and intervention durations should be structured to capture this period. The observed post-wash-out response likely reflects slower absorption and tissue redistribution of carotenoids, rather than a direct antioxidant effect mediated by omega-3 fatty acids.

Furthermore, the dissociation between carotenoids/serum L+Z and oxidative stress markers underscores the complexity of antioxidant biology and supports a complementary approach to characterising biological responses. Future studies employing longer intervention periods and larger cohorts are warranted to further elucidate the impact of combined omega-3 supplementation and L+Z intake on oxidative stress markers and long-term omega-3 incorporation into cell membranes. Also, adherence to the LCOD list would likely become a challenge for subjects if study periods were extended.

## Figures and Tables

**Table 1:** Participants’ characteristics (N = 16).

Characteristics	Men.	Women	*p*-Value	95% CI
N	10	6		
Age (Yrs.)	31.38 ± 5.07	30.50 ± 4.76	0.599	(−7.16, 4.36)
Weight (Kg)	75.07 ± 22.04	57.90 ± 11.8	0.004 *	(−44.81, −10.26)
Height (m)	1.70 ± 0.01	1.60 ± 0.04	0.000 *	(−0.22, −0.08)
BMI (Kg/m^2^)	25.42 ± 5.02	22.30 ± 3.81	0.037 *	(−9.78, −0.04)
Waist Circumference (cm)	88.00 ± 15.00	78.00 ± 10.00	0.027 *	(−29, −2)
Hip (cm)	107.00 ± 9.00	95.00 ± 11.00	0.048 *	(−23, −0)
Waist-to-Hip Ratio	0.86 ± 0.08	0.83 ± 0.09	0.298	(−0.15, −0.05)
Serum L+Z (µg/mL)	1.60 ± 1.59	0.87 ± 1.50	0.166	(−2.89, 0.56)
RBC Omega-3 Fatty Acids (%)	8.47 ± 2.74	7.65 ± 3.54	0.439	(−5.07, 2.44)
Serum MDA Levels (µmol/L)	0.67 ± 0.14	0.68 ± 0.09	0.849	(−0.12, 0.15)
Fat-Free Mass Index (FFMI) (Kg/m^2^)	18.02 ± 3.10	14.98 ± 1.35	0.000 *	(−6.80, −2.91)
Fat Mass Index (FMI) (Kg/m^2^)	7.39 ± 2.92	7.20 ± 2.99	0.903	(−3.62, 3.24)
Total Energy Expenditure (MJ/day) (TEE)	11.55 ± 2.40	9.26 ± 1.10	0.000 *	(−5.18, −2.15)
Physical Activity	1.65 ± 0.09	1.6 ± 0.08	0.566	(−0.13, 0.07)
Resting Energy Expenditure (MJ/day) (REE)	6.99 ± 1.34	5.65 ± 0.40	0.000 *	(−2.91, −1.37)
Visceral Adipose Tissue	2.73 ± 1.87	2.32 ± 2.10	0.540	(−2.97, 1.66)

* *p* < 0.05 = significant (independent sample *t*-test), CI = 95% Confidence Interval.

**Table 2:** Baseline characteristics of control and intervention groups.

Characteristics	Control (N = 9)	Interventions (N = 7)	*p*-Values	95% CI
Age (Yrs.)	34.11 ± 1.66	27.86 ± 0.88	0.009	(1.86, −10.65)
Serum L+Z (µg/mL)	2.15 ± 0.68	1.09 ± 0.51	0.260	(−0.87, −2.97)
MDA Level (µmol/L)	0.69 ± 0.06	0.65 ± 0.03	0.591	(−0.11, −0.19)
Omega-3 Fatty Acids (%)	8.69 ± 0.85	8.21 ± 1.19	0.750	(−2.60, −3.52)
Waist Circumference (cm)	91.00 ± 6.00	84.00 ± 3.00	0.331	(−8, −23)
Hip (cm)	107.00 ± 3.00	96.00 ± 4.00	0.293	(−7, −22)
FMI (Kg/m^2^)	8.30 ± 1.17	6.21 ± 0.55	0.038 *	(0.006, −0.22)
FFMI (Kg/m^2^)	18.76 ± 1.14	17.07 ± 0.96	0.296	(−1.65, −5.01)
Weight (Kg)	83.03 ± 8.30	64.84 ± 4.93	0.103	(−4.15, −40.5)
Height (m)	1.74 ± 0.03	1.66 ± 0.03	0.119	(−0.02, −0.17)
BMI (Kg/m^2^)	27.06 ± 1.92	23.32 ± 1.16	0.144	(−1.44, −8.93)
TEE (MJ/day)	12.10 ± 0.84	10.83 ± 0.78	0.298	(−1.25, −3.80)
Physical Activity	1.64 ± 0.03	1.66 ± 0.04	0.789	(−0.11, −0.09)
REE (MJ/day)	7.36 ± 0.50	6.51 ± 0.38	0.217	(−0.56, −2.27)
Visceral Adipose Tissue	2.87 ± 0.66	2.54 ± 0.71	0.746	(−1.77, −2.41)
Waist–Hip Ratio	0.84 ± 0.04	0.87 ± 0.02	0.563	(−0.12, −0.07)
% Dietary Adherence	84.39 ± 3.61	91.26 ± 2.90	0.177	(−17.23, −3.50)

* *p* < 0.05 = significant (independent sample *t*-test), CI = 95% Confidence Interval.

**Table 3:** Serum lutein+zeaxanthin (L+Z) concentration across study visits (control vs. intervention).

Study Day	Control (n = 9)	Intervention (n = 7)
Day 0 (µg/mL)	2.23 ± 0.56	2.49 ± 1.14
Day 7 (µg/mL)	2.23 ± 0.65	1.20 ± 0.53
Day 13 (µg/mL)	2.81 ± 0.90	2.63 ± 1.21
Day 19 (µg/mL)	2.98 ± 0.69	3.02 ± 1.27

Values are presented as mean ± SEM (standard error of the mean). The study visits correspond to Days 0, 7, 13 and 19.

**Table 4:** Observed absolute changes in serum lutein+zeaxanthin (L+Z) concentration across study periods.

Time Point	Control (n = 9) Mean Δ from Baseline (SEM), µg/mL	Intervention (n = 7) Mean Δ from Baseline (SEM), µg/mL
Baseline (Day 7 *)	0.00	0.00
Day 13 (Δ from Day 7)	+0.58	+1.43
Day 19 (Δ from day 13)	+0.17	+0.39

* Baseline defined as Day 7. Values are mean change (Δ) from baseline ± SEM (µg/mL); Day 19 values represent incremental changes from Day 13.

**Table 5:** Individual AUC and iAUC for control group (n = 9).

Participants ID	AUC 0–7 (µg·day/mL)	iAUC 0–7 (µg·day/mL)	AUC 7–13 (µg·day/mL)	iAUC 7–13 (µg·day/mL)	AUC 13–19 (µg·day/mL)	iAUC 13–19 (µg·day/mL)
1	5.50	0.00	3.33	0.27	4.08	0.48
2	36.40	6.93	44.64	7.50	47.31	0.00
3	23.38	1.26	16.59	0.00	10.08	0.00
4	14.70	5.39	12.42	0.00	16.92	9.30
5	25.72	0.00	19.83	0.00	20.22	1.38
6	22.02	0.00	18.21	7.65	25.92	0.06
7	2.70	0.46	14.28	11.58	20.04	0.00
8	4.38	0.00	1.80	0.00	2.85	1.65
9	5.49	0.53	4.98	0.00	9.09	4.29

**Table 6:** Individual AUC and iAUC for intervention group (n = 7).

Participants ID	AUC 0–7 (µg·day/mL)	iAUC 0–7 (µg·day/mL)	AUC 7–13 (µg·day/mL)	iAUC 7–13 (µg·day/mL)	AUC 13–19 (µg·day/mL)	iAUC 13–19 (µg·day/mL)
1	2.31	0.00	2.22	0.72	4.26	1.32
2	28.32	0.00	29.37	5.79	28.80	0.00
3	35.94	0.00	28.59	20.49	55.08	6.00
4	11.72	0.04	10.14	0.06	10.41	0.21
5	3.32	0.04	2.97	0.09	2.94	0.00
6	4.52	0.00	2.46	0.00	7.86	5.82
7	4.16	0.00	4.68	3.36	9.24	1.20

**Table 8:** Mean (±SEM) serum lutein + zeaxanthin (L+Z) concentrations during wash-out and post-wash-out.

Study Time Point	Phase	Control (n = 9) Mean ± SEM (µg/mL)	Intervention (n = 7) Mean ± SEM (µg/mL)
Day 0	Wash-out	2.23 ± 0.56	2.49 ± 1.14
Day 7	Wash-out	2.23 ± 0.65	1.20 ± 0.53
Day 13	Post-wash-out	2.81 ± 0.90	2.63 ± 1.21
Day 19	Post-wash-out	2.98 ± 0.69	3.02 ± 1.27

**Table 9:** Changes in MDA and omega-3 PUFA biomarkers following intervention compared with control.

Variable	Group	Baseline (D0–D7) (Mean ± SEM)	Post-Intervention (Day 19) (Mean ± SEM)	Absolute Change
Serum MDA Levels (µmol/L)	Control (N = 9)	0.69 (0.06)	0.66 (0.03)	−0.03
	Intervention (N = 7)	0.65 (0.03)	0.74 (0.06)	+0.09
RBC Omega-3PUFAs	Control (N = 9)	8.68 (0.85)	9.89 (1.16)	+1.21
	Intervention (N = 7)	8.21 (1.19)	8.69 (1.96)	+0.48

**Table 10:** Pearson’s correlation coefficient between baseline serum lutein + zeaxanthin (L+Z), dietary adherence, and malondialdehyde (MDA) levels at Day 7.

Variable	N	R-Value	*p*-Value
Serum L+Z levels vs. MDA levels (Day 7)	16	0.529	0.035 *
% Dietary adherence vs. MDA levels (Day 7)	16	0.550	0.027 *

Pearson’s correlation coefficient^®^ reported. Day 7 corresponds to the post-wash-out assessment timepoint. * *p* < 0.05 = significant (2-tailed).

## Data Availability

The data supporting these findings are available in the UNSW data archives at: https://www.unsw.edu.au/research/data-archive (accessed on 11 December 2022).
